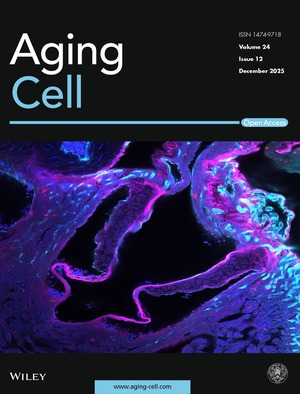# Additional Cover

**DOI:** 10.1111/acel.70321

**Published:** 2025-12-08

**Authors:** Laura Bevan, Jessica Radford, Helena Urquijo, Joseph Carr, Alice Etheridge, Stephen Cross, Melanie Hezzell, Rebecca J. Richardson

## Abstract

Cover legend: The cover image is based on the article *Aged Zebrafish as a Spontaneous Model of Cardiac Valvular Disease* by Laura Bevan et al., https://doi.org/10.1111/acel.70266.